# Croton Oil Plaster

**Published:** 1845-03

**Authors:** 


*Croton Oil Plaster.*—M. Bouchardat recommends the following method of preparing croton oil plaster. Melt eighty parts of gum diachylon plaster at a very gentle fire, and when it is semi-liquid, mix with it twenty parts of croton oil. The plaster which results is to be spread thickly on muslin. It will produce considerable irritation of the skin, and may be employed in all cases where revulsives are required. It does not cause such severe pain as many other counter-irritants; and it may be applied over an extensive surface, so that a derivative action may be established proportional to the irritation which is to be combated,—an indispensable condition in the employment of these heroic remedies. M. B. is fully of opinion that the croton oil plaster will be found available in the treatment of many chronic diseases, both of the respiratory apparatus, and of the abdominal viscera.—*Annuaire de Therapeutique.*

